# On Cooling of the Body after Death

**Published:** 1863-01

**Authors:** Benjamin W. Richardson

**Affiliations:** Senior Physician to the Royal Infirmary for Diseases of the Chest.


### Art. II.—ON COOLING OF THE BODY AFTER DEATH.

By Benjamin W. Richardson, M.A., M.D.

Senior Physician to the Royal Infirmary for Diseases of the Chest.

I have been requested to put on paper a few facts relative to the cooling of the body after death. The subject, of interest generally, has become of special importance owing to the late trial, for murder, of a man named Gardner. This man was found guilty and doomed to be publicly strangled. His fate turned exclusively on the vague question whether a dead body could become cold on the external surfaces of the head, chest, and upper and lower limbs, within three or from that to four hours. A negative answer to this question led to his conviction, and the man was so decisively condemned that the judge told him not to hope for mercy. But when the trial was over, the Secretary of State, Sir George Grey, received from medical men so many facts in opposition to the opinion that was expressed at the trial, that he had to reverse the judgment, and to spare the culprit's life.

The profession asks, and the public asks, how is it possible that such difference of opinion could exist? And I believe there is not one thinking person who did not feel anxiety for the Home Secretary, in the dilemma in which he was cast. Happily in cases such as that of Gardner, there are these indications as guides to the official mind; that where there is doubt it is always safe to err on the side of mercy, and that should an error be committed on the side of mercy, none except the professional sight-goers to executions can be any the worse for it, and they not really the worse, but something the better, in regard to their influence on the world, from their own small but vicious centres of power.

I hope that never again the life of a human being will be allowed to rest on the question of the time required for the body of a dead person to become what is called "*cold.*" It is a blot both on our national character for knowledge, and on our humanity, that such a hazard has ever been played. If the fire of a steam-engine had suddenly gone out, instead of the fire of life from a living body, and an engineer had risen in the witness-box and sworn that because certain external parts of the engine were cold, therefore the engine had stopped play at least three hours; and if on that evidence the life of a man had been condemned by a judge and jury, the public would have pronounced judge and jury as madmen. The evidence and its results would have been considered simply atrocious. Yet, in truth, in the Gardner case, this is what was done; done and tolerated because the people are struck with a kind of mysterious influence whenever the living mechanism is considered and commented on. The expositor of the steam-engine is a man, he must speak cautiously; the expositor of the physics of the body is an oracle, what he says must be believed because there are none but men of his own class to correct him. It is not to be assumed that this condition of things will last. In time the people will learn the laws of life as they now learn arithmetic, geography, or other common branches of knowledge, then anomalies and mysteries will disappear.

I repeat, that after what has occurred in this case of Gardner, it is impossible that any man will ever again be tossed over to the executioner on the speculation that a body must have been dead *so long* because it was *so cold.* Nevertheless the question of the cooling of the body is an interesting one, and one on which but little has been said. I regret that to what has to come in this paper, much more might be added; but at all events, that which is given will supply certain approximations to the truth, together with some positive facts and deductions from them.

The first point to be remembered in respect to the cooling of the body is, that as regards the body altogether, the cooling process, as a general rule, goes on after death without any movement in the opposite direction. But this rule is subjected to a rare exception. Exposed surfaces of the body, such as the cheeks, left with a capillary network full of blood, may become coloured after death and even slightly warm. In a man who died from suffocation in the village of Broughton Astley, near Leicester, about fourteen years ago, and whom I endeavoured to restore by long continued inflation of the chest, this appearance was at one time so striking that the bystanders believed fully the man was being restored. His cheeks became red; his lips red; and in the lip-muscles there was the slightest appreciable quivering movement. This latter effect continued probably fifteen minutes, and the redness much longer. The cheek, previously cold and deathly, became also warm to the touch. These effects might be esteemed as due to the attempts that were made to restore animation; but this supposition must be eliminated, because I have seen the same phenomena in cases where no attempt to restore life had been made. Dr. Snow was once called to see a young woman who, after having been dead three days, suddenly became so suffused and red, that her friends doubted the fact of her death. After a time, however, the colour abated; and commencing putrefaction proclaimed that the dissolution was perfect. The delusive signs thus given have a very simple explanation. The coloration takes place only in parts where there is a wide surface of capillary vessels, and a surface which is superficial as well as wide, and exposed to the air, such as the centre of the cheeks and the lips. Into such a surface of vessels left charged with blood, the external oxygen finds its way; and a certain amount of combination takes place between the oxygen and blood, attended with evolution of heat, with vivification of colour, and, if muscles be near to be influenced by the calorific ray, with slight uneasy movements in such muscles.

I name the possibility of return of warmth in a dead body because, rare as it is, it might lead to serious misapprehensions. A case might occur in which a body that had been dead over forty-eight hours was found with the cheeks flushed and warm. Assume a medical witness, taking superficial note of this fact, and swearing thereon that the body was but just dead or had not been dead more than one, two, or three hours, because the face was warm! such evidence bearing suspiciously on the conduct of any prisoner might tell against him even more readily than the opposite statement, that a body had been dead so long because it was cold.

The temperature of the dead body, placed in the air and resting on a non-conducting surface, sinks from the moment of death. The process of cooling then continues until the temperature is reduced to that of the surrounding air, at which point it stops. But if, after the body has reached the minimum temperature of the air, the air rises in temperature, the body still remains at the minimum temperature until putrefaction sets in. The dead body is thus, for several hours after death, like a minimum thermometer marking the lowest degree of heat that has occurred. For instance, if death take place with the air at 60° Fahrenheit, and a dead body is placed on a non-conducting substance, such as a blanket, the temperature of the body will fall to 60°. But if the temperature of the air afterwards rise to 70° or 80° the body will still register 60° until it begins to decompose.

It is not always, however, that the temperature of the body falls suddenly to one point and remains there. It will follow the air to a certain point, and remain fixed while the air rises: but the air may fall again below 60°, to 50° to 40°. Then the body continues to follow, and goes to the minimum, there to remain, whatever may be the temperature of the atmospheric medium afterwards, *i.e.*, to whatever height that medium may rise in a natural way. The same rule that applies to the air, applies within temperatures varying from 40° Fahrenheit to 96° to other substances by which the body may be surrounded. Thus, if a warm blooded animal is drowned in water having a temperature of 96°, and if the water is retained at that temperature, the body keeps its caloric; if the temperature in which the animal is drowned be below 96° (say, again, 60°), the body of the animal falls to that temperature and remains at that: taken out and plunged into water at 96° it gains only superficial warmth: to raise the temperature of the mass, the water must be injected into the arteries, and even then the heat must not be lower than 115°, to enable it to bring the temperature of the whole to 96°. The general law, then, applicable to the cooling of the dead body under ordinary circumstances is, that the temperature of the body falls to the surrounding medium, whatever that may be; but that the temperature of the body does not rise with the mere increase of temperature of the surrounding medium.

There is one other general rule which deserves notice, and which, I believe, is absolute; viz., that *cæteris paribus*, the rate of cooling in a medium colder than the body, is the same in all cases, down to the temperature of the surrounding medium. Thus, if two animals are destroyed at the same time, in the same way, and under the same conditions, and if one is placed in a temperature of 60°, and the other in a temperature of 40°, both animals will give a temperature of 60° at the same time when the one in the water at 60° has reached its minimum; but in the case of the animal that is in the lower temperature, the cooling will continue down to 40° before the equilibrium is determined.

These facts in regard to cooling of the animal body are very simple, and by comparison in analogous circumstances admit of being reduced to formulæ; but as there are no two bodies absolutely alike, as there are no two bodies left after death in the same conditions absolutely, and as no two bodies die precisely in the same way, the rate of cooling of any dead body is subject to great variations, of which we can only form approximative measurements.

There are three grand modifying circumstances to be considered on this subject:—

1. The condition of the body itself.
2. The surroundings of the body.
3. The mode of death.

In regard to the condition of the body itself, even in cases of sudden death, unattended by organic changes, considerable variations, dependent on the natural external warmth of the body during life, are presented. It is a fact not easily explained, but still true, that in some persons the temperature of the skin naturally is below 96°, and is, therefore, by comparison, to a hand at 96°, cold. One cannot shake hands with half-a-dozen persons half-a-dozen times without becoming conscious that there are gradations of temperature natural to the hands of these persons, one's own hand being the standard; but when the hand is thus naturally cold, all exposed parts are equally cold; not only so, persons naturally cold, if they go lightly covered, are soon seized with general chillness. They instinctively court warmth—live on it, luxuriate in it. In summer time they themselves lose the subjective consciousness of cold, but to another person of warmer temperament they present still the objective or comparative idea of coldness. If they die from any sudden cause, the fact of coldness could be no test of the time of death. If in parts warmly covered they were warm without redness, (the exceptional phenomenon of revivification of the cutaneous surface being remembered) it might be inferred, from a knowledge of the previous history of the person, that death had not long taken place, but even then no boundary of time could be safely marked out.

The condition of the body in respect to the amount of fat makes a great difference. In men with large chests and no extreme lankiness, in whom the heart has a short surface to irrigate, in these men caloric is abundantly produced, nutrition is active, more fatty food is often taken in than can be burned, the cellular tissue becomes the store-house of non-conducting adipose material, and warmth is long retained. But even in these cases, if the body is left uncovered, the external surface cools with moderate quickness. I believe the external warmth rarely extends beyond fifteen hours. I once made the post-mortem of a plethoric man, who died suddenly in a convulsive paroxysm while sitting up in bed. He was a violent man, and in a fit of rage he threw some article of clothing at his attendant, was seized directly with spasm of the chest, and died before assistance could be procured. Thirteen hours afterwards I conducted the post-mortem. There had been no loss of blood from rupture of vessel, and both sides of the heart were full of blood; the body also had been covered, and a fire had been in the room for some part of the time, but the body was cold externally at every part, though not rigid. On opening the visceral cavities, however, the intestines and large organs still retained warmth, to my sensation; they were warm to my hand, which was of its usual warmth. I name this case, because it is one in which every circumstance tended to favour the retention of heat by the external surface, and yet the external cooling was well marked thirteen hours after death.

Persons who are naturally very thin, cool after death with great rapidity, whatever may be the form of death. It requires but slight observation of disease to recognise the truth of this fact. I have seen patients with phthisis pass before me in the heat of summer, and as pulse after pulse was taken, I have felt the antecedent coldness of death in the moist chilly hand. I have seen such die, and have known no difference between the temperature of the external surface of the body before and after the fatal event. In common anæmia the same extreme coldness prevails; in anasarca also, and to a certain extent in diabetes, uræmia, and ague, to say nothing of cyanosis.

Periods of life make a difference in the cooling of the body after death: in the infant and the aged person the process is extremely rapid. In the aged, who die the natural death by sleep, the cooling is so gradual before the death that it is difficult in some instances to tell from the mere temperature whether the body is alive or dead, I saw an instance of this kind recently, where the somnolency of dissolution, senile coma, continued for a period of three weeks, and in the last hours so deeply tranquil was the repose, so cold the body, despite all attempts at supplying artificial warmth, that it required a watchful eye over the respiratory movements to say "life has not ceased." So, during certain conditions in adults, where blood is diverted from the system to answer some second or supplementary purpose, the same chilliness prevails. Anæmic thin women, in the last stages of pregnancy, are often unnaturally chilly, and labour is preceded by universal surface coldness. I remember a case when I was in general practice, where a woman in her eighth month of pregnancy died suddenly from syncope. I was at some distance, but was speedily summoned, and was on the spot within an hour and a half after the death; the patient was robed in the white pall of mortality; she was as marble, and the fingers were rigid; her body universally cold.

I made also the post-mortem examination of a lady who died in the same sudden way, during the seventh month of pregnancy, in the year 1860. This lady had appeared well up to the moment of her death. She had been out to the shops in the morning to order the household necessaries; she had returned and was playing at ball with a child when she suddenly reclined on a dresser, then fell, and was picked up dead. Dr., now Professor, Halford was called in and attended instantly, but found life quite extinct. The friends told me that the event was so sudden they could not realize it as death, but that the rapid and deathly coldness of the body first aroused their fears. "Before the doctor came," to use the expression of one of the bystanders, "she was like a stone, she was so icy cold, so that I felt sure she was dead." We found that in this case death had been produced by the liberation of a fibrinous plug into the pulmonary artery, and by mechanical obstruction of the circulation.

The coverings of the body and the character of the surface on which the body rests after death, exert a material but not easily calculated influence on the process of cooling. If the body recline on a cold and absorbing surface, such as a brick or stone floor, the cooling will necessarily take place with greater rapidity than on a bed of straw or feathers; so again a body unprotected from the external air will seek the temperature of the air more quickly than if it were surrounded with non-conducting material. I thought once that some rule might be arrived at on this point by taking the temperature of sheep after death by the knife, and by comparing the decline of temperature of a sheep that was shorn with another that was not shorn, both being killed at the same time. I made the observation with two small bulb thermometers by placing a thermometer in each case beneath the shoulder of the animal, that being a point where the skin is most free from wool. The results I obtained were, at least, interesting. I found that for the first twenty-six minutes after death the thermometer fell half as slowly again in the animal that was warmly covered, compared with the one that had lost its non-conducting envelope; but after this period the change was scarcely perceptible. I have tried to carry out the same inquiry in small animals recently dead by covering them with flannel of various degrees of thickness; but the results are so variable that I can reduce them to no rule, and the only inference I can draw in respect to the human subject is, that for the first two hours the body covered with bedclothes would retain its warmth in parts such as the abdomen and flexures of the limbs, but that beyond that time the loss of external temperature is so decided and universal as to render any opinion mere vague conjecture altogether inadmissible in the way of evidence.

We come now to the most important modifying cause, I mean mode of death. The great rule in respect to this is comprised in saying, that the rapidity of the cooling process turns greatly on the point, whether the circulation fails prior to the respiration; or, to put the matter more definitely—whether the body is left full of blood heated to the ordinary degree, or either empty of blood, or charged with a blood the oxidation of which has been, for some time previously to death, partially suspended. In all cases where the blood is suddenly removed from the circulating channels, the decrease of temperature is immediate. The temperature of the solids is, in fact, so sudden upon the withdrawal of the blood from them, that the actual period of cooling may be said to be determined by the rapidity of the withdrawal. We see the immediate effects of withdrawal of blood in the ordinary act of fainting: we see it still better in hæmorrhage, either internal or external. The decline of the temperature in these cases is so great, that the external surface of the body may actually run down to that of the air without death. In a case of uterine hæmorrhage to which I was once summoned, the body was so unusually cold that a thermometer placed under the tongue gave the same degree as the external air. Still there was faint breathing, and the vagina and os uteri being carefully plugged, and stimulants gradually and cautiously given, recovery took place, and that patient is now alive and well. I observed the same fact in a case of epistaxis in a child. Further, in examples of death from fibrinous deposition in the right heart, where the blood is not removed from the body, but is simply arrested, from the obtruction, in its circuit through the lungs, the body may be reduced to the temperature of the surrounding air even before death. In one of my earliest papers on the symptoms of fibrinous deposition, I commented on this marble coldness of the body antecedent to death, as one of the best diagnostic indications of the cause of the sinking presented by the patient. I have seen this coldness of surface in as many as twenty-six examples of sinking from fibrinous deposition; and have pointed out that whenever, in an acute inflammatory attack, there is intimation that the feet cannot be kept warm, the first warning is given of impending arrested circulation and death.

In cases of sudden death from effusion of blood, where even the blood is not lost from the body, the cooling process may be perfected so quickly that the suspicion of death having taken place some hours might occur to one who was not familiar with all the facts. I had once under my care at Mortlake, a thin, pale, anæmic girl, with a large and flabby heart. She worked in a laundry, the temperature of which was always high. This girl was subject to frequent attacks of syncope. One day while at her work she fainted, as it was believed, but her friends being unable to restore her by holding sal volatile to her nose, and by using brisk friction, sent for me. Some half-hour had elapsed when I arrived, and I at once saw that she was dead. She was cold universally, and white as the sheet on which she lay. While I was present rigidity actually commenced in the limbs. Had not the fact been indisputable that this girl was working at the iron within the hour, I should, at that time, have guessed that she had been dead three or even four hours. The determining cause of the fatal result was effusion of blood into the lungs.

I have by me the notes of another case, where a gentleman, apparently in his usual health, rose from the breakfast-table and went to the water-closet. He remained there so long that his relatives became anxious and called to him. He did not answer, and the door therefore was forced. The man was dead, cold, and partly rigid; he had been in the water-closet some time less than an hour. At the period when this event happened the last cholera epidemic was showing itself, and under the circumstances, owing to the place in which the death happened, and the choleraic aspect of the body, the inference was jumped at that the man had fallen a victim to the dreaded disease; and so a post-mortem was made, and lo! the death was found to have occurred from rupture of an aneurism and effusion of blood into the pericardium.

Cases of death from wounds and hæmorrhage are happily so rare in the human subject, that we have few opportunities of determining the period at which the body cools after them. I have tried to gather some kind of information in this direction by researches on inferior animals at the slaughter-house; but as there are many circumstances in the way to retard the cooling process there, it is not easy to define the rate of cooling. In a shorn sheep, I determined, on a day the temperature of which was 65° Fahrenheit, that under the shoulder the thermometer, which before the death of the animal stood at 99°, fell to 79° in thirty minutes. At this time the mercury was beginning to fall more rapidly, and the body of the animal was everywhere cold to my hand—even the tongue and inner surfaces of the mouth were cold, but I was obliged to give up the observation, owing to the fact that the coldness interfered with the process of dressing. Afterwards I made other observations, the results of which all centred round the first experiment, and I believe it is not far from a correct definition of the cooling of an animal body. The mean temperature of the sheep is 2° above that of the human body, according to my estimate, and the process of cooling would be somewhat slower in the sheep, other things being the same. But taking these differences out of the argument, and placing the cooling of a human subject that had died from sheer and sudden loss of blood, in comparison with an inferior warm-blooded animal that had died from the same cause, we may fairly estimate that with the temperature of the air at 65°, the body exposed to the air, or protected only by linen clothing, would sink to 76° in twenty minutes, and to the temperature of the air in forty-five, or at most fifty minutes.

But as I wish to put every difficulty forward, I must state that here again differences may be presented due to causes which at first sight may not appear; for instance, the question of the last meal makes a difference in the cooling process; if the body is fasting at the time when blood is withdrawn to the death, the cooling is much quicker than during the assimilation of a hearty meal. Intelligent butchers know this fact well and act upon it: if they wish a quick cooling, they kill their animals fasting.

It is not necessary that the blood in the mass should be lost from the body to ensure rapid cooling; it is enough that the watery part should drain away. How well marked is this fact in cholera, I need not explain. I believe that if I were taken blindfolded to a patient in the collapse of cholera I could diagnose the disease by the touch alone: so cold, so shrivelled, the skin of the sufferer by the side of that of the healthy man. And how rapid these changes! A man named Dade, in 1854, was in my consulting-room at ten in the morning, complaining only of slight pain in the bowels; his skin then was warm to my touch; at eleven and a half, he was in bed, thickly covered, with bottles of hot water around him wherever they could be conveniently placed, and yet the thermometer under his tongue registered but 68°, his breath fell cold on my hand, and his face (for the man might have sat for the picture), carried me instinctively, and in spite of all surroundings, to the rising Lazarus of Haydon. All this within two hours: a living body wrecked by a sudden blast; its fire choked, its engine power out of gear, its governing intelligence unable to command, and its movements, such as they were, wild and pitiful! A chaotic wreck of life, drifting on the shores of death in a storm of two hours! And yet that wreck might recover, as it did in the case of Dade. Had it not recovered, the temperature after the death would have remained scarcely modified from that which immediately preceded the dissolution.

There are some poisons which produce a form of death, followed by very rapid cooling: the cyanides are of this class, oxalic acid, and bichloride of mercury. All these, when they kill quickly, destroy the heat-producing force of the blood, and effects analogous to those arising from hæmorrhage, and its ally, choleraic flux, are the natural sequences.

To sum up: the knowledge we possess as to the cooling of the body after death may be included all in the following heads:

1. If the body is left dead with its vessels full of blood, the temperature of the blood being unaffected by the mode of death, the cooling is slow, but in the great majority of cases is completed in fifteen hours.

2. If the body is left dead from direct and absolute loss of blood, cooling to the temperature of the surrounding medium is completed, in regard to the external surface, in two hours.

3. If the body is left dead from sudden and profuse exudation, as in cholera, the cooling to the temperature of the surrounding medium is completed in two hours.

4. If the body is left dead from obstruction to the circulation, as from fibrinous concretion, the body, in so far as the external surface is concerned, will be chilled to the temperature of its surrounding medium in two hours.

5. In these last three named forms of death, if the death be slow, the heat of surface may sink to that of the surrounding medium, even before life has ceased.

6. The body, when dead, will sink steadily in temperature to the medium of its surrounding envelope: in the air to the air, in a stone tomb to the tomb; but it will not afterwards rise in temperature by the application of any external warmth, short of such as would destroy its texture.

7. After all forms of death, the age and corporeal condition of the person must be taken into account; youth and old age, great thinness of structure, deficiency of food, and states in which blood is diverted from its systemic course, quicken the decline of the animal heat.

8. In taking observations for medico-legal purposes, in any suspected case, the mere test of the hand is altogether unreliable: for as the terms heat and cold are relative only, and as between the hands of different observers the greatest natural difference may prevail,—that which to one hand would signify warmth, to another would signify cold. If any observation in respect to temperature be made, therefore, it should be carried out with the thermometer, the points at which the temperature is taken being the flexures of joints, the mouth, or the nostril.

In conclusion I have to offer but one other observation. In all cases the temperature of the air should be taken with care, and a comparison should be instituted between the body and the air. By such a comparison, in any case where the body had been left lying on a non-conducting substance, such as a bed, and where there was no evidence that the surrounding air had been raised in temperature by artificial means, certain facts might possibly be made out in some instances, by a strict analysis, conducted in the following manner. Compare the temperature of the body with that of the air ; if the temperature in several parts is above the temperature of the air, the inference is strong that the body has not been dead two hours. If its temperature be the same as the air, the inference is fair that the body has cooled to that temperature within the period that the air has registered the said temperature. If the body is below the temperature of the air, the inference would be that it had cooled at a time when the air was at a lower temperature than existed at the period of observation ; and if then, on referring to the minimum self-registering thermometer, the observer could say at such an hour before the observation on this body, the thermometer registered in the air a minimum degree, of say 40°, and this body registers 40°, the proof would be conclusive that the said body had been surrounded by a medium of 40° after its death. The inference would thereupon fairly follow that the said body was dead at the hour when the thermometer had reached its minimum.

It may be that in time some more accurate facts will be made known, and some ingenious instrument be devised for measuring the process of cooling after dissolution. If I had time, I could, I think, produce an instrument for carrying on careful researches of the nature suggested ; but the question involved is really of little moment if the medical community will only be cautious in giving opinions upon it once or twice in a century. I have shown a few facts, and many sources of doubt in the above history, and I cannot end better than by remarking, that although I have perhaps looked into the matter as carefully as any other physician or physiologist of my day, I would sooner cut off my own right hand than send any human being to the scaffold on a dogmatic statement based upon the hypothesis that the period of death can be determined by the temperature of the dead.

